# Medical Miscellanies

**Published:** 1816-05

**Authors:** 


### PART IV.

### MEDICAL MISCELLANIES.

### DR. SEEDS ON BLOOD-LETTING.

(Continued from page 90.)

*4th Experiment.* The carotid artery of a very small dog was opened in the middle of the neck; the blood rushed impetuously to the distance of several feet. In two minutes, the strength of the animal sunk, nor did it ever recover vigour.

The motions of the heart though feeble, were regular; the breathing was hurried, and somewhat irregular.

For a considerable time before death, the body was alternately bent and contracted, in a manner resembling shivering; shortly before death, the breathing became hoarse and laborious.

The cries of the dog were at first shrill, but gradually became feeble; and at last a feeble howl only was uttered: the face and limbs speedily became cold; in ten minutes life ceased. The limbs at this time were flaccid and flexile.

The viscera of the abdomen appeared plentifully supplied with both red and blue vessels. The small intestines were somewhat contracted, and contained three or four lumbrici. The urinary bladder was rugous, and empty. The vena cava was pretty full; and some small dark clots were found in the aorta.

The lungs when inflated were white ; but when collapsed, they appeared reddish. A small quantity of blood was found in most of the large veins.

The dura mater of the brain was colourless and transparent, except at the falx, where there was a red spot, and some slight adhesions.

The lateral ventricles contained a great quantity of water. The plexus choroides was pale; a few red vessels were seen amongst the convolutions, and one blue one distended on the corpus callosum. The nerves were colourless, except the optics, round whose origins a few red vessels were seen.

The sinuses were quite bloodless. As for the spinal marrow, its tunics were rather red at the top and bottom. No venous blood was to be seen in any part of the vertebral column.

Bichat asserts, that he has found by experiment, that, the curvatures of vessels do not impede the motion of the blood ; but we are induced to suppose he might have erred, for, on opening the carotid artery, a great quantity of blood was immediately lost, and the animal was, as it were, destroyed at one stroke. It was not so when the *subclavian* was opened ; and this vessel though more curved, is not more distant from the heart.

Bichat when investigating this subject, chose for the objects of experiment, vessels remote from the heart, as those of the intestines. As we shall see afterwards, a much greater quantity of blood was lost from the carotid when opened, than from the aorta itself. Blood was deficient in most of the organs; the nerves were for the most part, moist and bloodless.

On the opening of arteries, dark or venous coloured blood almost always abandoned the brain. What reason can be assigned for this fact?

*5th Experiment.* All the larger veins of the legs were opened in a small dog. At first the pulse was accelerated ; soon after it became slow and languid. The heart's motions though feeble were never irregular ; and indeed, long before death, they could neither be seen nor felt. Borborygmi were early heard, and lasted a long time. The breathing at first was hurried ; soon it became slow and laborious, at last convulsive. The pupils were frequently examined; they became gradually less and less obedient to the influence of light, and at length ceased to contract altogether. Slight spasmodic contractions took place, first in the femoral and abdominal muscles, then the head, neck, and fore legs, were likewise powerfully affected with spasms.

At this time a deep sleep seized the animal; he breathed slowly and with difficulty, and for a little time before death, respiration, at intervals, was suspended altogether. Whenever the breathing was strong and quick, the pupils recovered their tone, and the blood was more strongly propelled. In an hour death closed the scene.

Some degree of stiffness was apparent in the legs, especially the fore ones.

The dissection of the head was first begun. The membranes of the brain were *loaded with turgid vessels,* the larger of which, were of a very dark colour.

A bright red spot was observed near the cornua, where some degree of sanguineous effusion had taken place. The sinuses were full of blood.

In all the ventricles there was more or less water effused ; the base of the brain, and the eighth and ninth pairs of nerves, were inundated with water ; a net work of red vessels was spread round their origins, and the optics were in the same state. In the cervical and lumbar regions of the spinal marrow, there was a considerable degree of redness. The right side of the heart was full of blood ; the left auricle contained a little; some blood was found in the larger veins; and a few clots in the thoracic aorta.

The stomach and all the intestines were tumid with flatus. The veins of the mesentery were turgid. The larger veins of both legs being opened, although life was not speedily destroyed, yet it quickly brought on the greatest degree of debility ; the slow weak motion of the heart, the languid countenance, the disturbance of all the functions, the apoplectic stupor under which he laboured, all conspire in proving this.

The turgid state of the *veins* of the head was very remarkable; indeed, throughout the whole body the *veins* were tumid.

Flatulent distentions of the stomach and bowels frequently distress the enfeebled; and it is well known that they result from excessive evacuations; all aged persons, those labouring under constipation or too great laxness of the bowels, or a hæmorrhage of any kind, are subject to this distressing affection.

*6th Experiment.* A healthy young dog was the subject of this experiment. On laying bare the internal jugular veins, we remarked, that they swelled much during expiration, and vice versa.

On freely opening these vessels, the blood burst forth with impetuosity ; and the animal's strength sunk almost immediately. They had scarcely been opened, when the heart beat strongly and sharply ; and a severe panting came on.

The head, neck, and back, were affected with powerful spasms. After two minutes, the pupils ceased to contract; and the eyes were twisted towards the nose. In about ten minutes he died.

The lungs were white with different coloured spots; a large black spot was also observed in the diaphragm.

All the *veins* of the head, neck, and chest, were tumid; moreover, the aorta and its larger branches were slightly stained with blood.

In several of the viscera of the abdomen, there were black patches ; particularly in the liver and spleen : and in these places, they were very weak, and easily torn.

The whole of the intestinal tube, as well as the stomach, was full of flatus. A little blood was found in the mesentery and omentum. The appearance of the brain was very similar to what it was observed to be in the last experiment; a greater number of distended *veins* were seen, and every thing was overwhelmed with water.

Round the root of the fourth pair was spread a plexus of vessels, and in a less degree round the eighth and ninth pairs.

In every sinus there were clots of dark blood. The tunics of the spinal cord were very red; especially the dura mater. On almost all the spinal nerves there was some degree of rubescence. The sinus venosi contained some clotted blood.

Why did this animal perish so rapidly, as if struck by lightening? The event was not at all in the ratio of the blood lost. All the symptoms clearly showed, that the brain was immediately *compressed;* which conclusion the anatomical examination of the body confirmed.

The contents of the cranium and spinal canal were so gorged with blood, that it might at first have been imagined that blood-letting would have saved the animal.

It does not seem out of place to remark here, that in every animal destroyed by blood-letting, and examined by us, we always found more or less of serous effusion in the brain, cerebellum, spinal marrow, and at the origin of the nerves; thus we clearly see why those examining the contents of the cranium have so frequently observed very turgid vessels and water effused, and likewise why venæsection has, in apoplexies, so frequently disappointed the expectations formed of it.*

*7th Experiment.* The right jugular vein of a young dog was divided; the flow of blood was copious and rapid for ten minutes, afterwards it became slow; the respiration was greatly hurried, so that he breathed 114, 120, 150, and at length 180 times in a minute.

At these times the discharge of blood was either scanty or ceased altogether, and the breath became cold; but when respiration was less hurried, the pulse became more regular, and the breath warmer. The upper orifice ceased to discharge blood before the lower. The heart's beats were at first unusually quick, soon they became feeble and slow, and sometimes were imperceptible.

In little more than half an hour we cut the vein again, and cleared it of a clot of blood; this appeared to give no pain. The eye sight soon became obtuse, and not long before death the pupils were immoveable. Almost the whole body was twisted by spasms. After eighty minutes had elapsed, *no signs of life were visible.*

---

* The above appearances certainly could hardly be expected *a priori* from theory: but we are of opinion, that although they were produced, without doubt, by *venæsectio ad mortem;* yet that a state the very *reverse* is produced by *moderate* venæsection, and in the early stage of this extreme blood-letting. The above experiments, however, elucidate the fatal effects of carrying blood-letting *beyond a certain point,* inasmuch as we then produce what we are anxious to prevent — congestion and effusion. Edit.

On examining the dead body, we found the whole of it stiff and twisted. The intestines were much swollen with flatus, the rectum and urinary bladder were contracted and rugous. All the veins of the thorax and abdomen were tumid. The right side of the heart contained a good deal of fluid blood, whilst a little was found in the left side and in the aorta. On the membranes of the brain, a wonderfully great number of veins was seen. A great deal of water was every where effused. Most of the nerves were pale; the eighth pair exhibited the same appearance as in the last experiment. The sinuses were full of thin watery blood. The membranes of the spinal marrow were plentifully supplied with red and blue vessels; the spinal nerves were covered at their roots with red vessels. At the same time that the breathing of this animal became very rapid and frigid, the flow of blood ceased, and the whole body became cold.

All the moderns, with one consent, agree, that the temperature of animals does not solely depend on the chemical action of the blood in the lungs; but that the powers of life, in some unknown manner, connected with the nerves and nervous system, over-rule and direct all the functions of living animals. What reason can be assigned for the superior orifice ceasing to discharge before the lower?

The loss of sensibility was proportioned to the loss of blood.— When much blood has been lost, or when it has been vitiated, the effects of all stimulants are very much diminished: many facts prove this.

Let a little of any active substance, as alcohol, opium, or digitalis, be given to a person faint from the loss of blood, what do we see happen?

The pulsation of the heart and arteries are slowly and slightly increased, and a gentle degree of heat is slowly diffused over the whole body. On repeating the remedy, the colour, from being changeable, becomes constant. The same may be said of a person labouring under severe typhus fever.

Who is ignorant that if a healthy person should take these substances, the result would be widely different?

Why was the death so tardy in this instance?

*8th Experiment.* The abdominal aorta of a small dog was nearly divided.

On the left parietes of the abdomen being cut, all the more moveable intestines protruded. The vessel was opened at about half an inch from the commencement of the iliac arteries.

The blood flowed with great force; the motions of the heart immediately became weak, but remained regular till the termination of existence. The breathing, at the same time, was slow and heavy; and was effected with opened mouth and the head thrown backwards, as if the animal panted for breath.

In four minutes time the pupils were dilated, and immoveable; the eyes, however, still retained their splendour. In six minutes he expired.—We first of all examined the viscera of the abdomen.

The intestines appeared plentifully supplied both with red and blue blood; the veins of the liver contained clotted blood. In both sides of the heart coagulated blood was found, in greatest quantity in the left, as likewise in the aorta descendens and vena cava; most conspicuously, however, in the former. The lungs, especially when inflated, were pale; their veins, and likewise the thoracic, were far from empty. The dura mater cerebri was moist, and some few red vessels were ramified through it. Vessels, red and blue, were conspicuous in the other tunics; the plexus choroides was pale; the ventricles were moist, the fourth was full of serum, a good deal of which was effused round the optics, the accessories, and the eighth pair. The greater sinuses were full. The larger vessels were of a dark colour; while the smaller, and those surrounding the origins of the nerves, were red.

The dura mater of the spinal cord had nearly the same appearance as that of the cerebrum. On removing this tunic, at the top of the medulla, there was a great deal of bright redness; a less degree of it lower down, and at the bottom some larger and darker vessels were seen. There was some degree of effusion within the dura mater, especially at the bottom of the cord.

The nerves were, for the most part, colourless. The fatty tunic was dark coloured, and the sinus venosi contained a little watery blood. Here the strength was quickly and equably broken.

Why was the left side of the heart, as well as the aorta, near the part cut, so full of clotted blood? It is well known, that when any thing opposes the motion of the heart, its parietes are, in the same ratio, thickened; but in this experiment, we see the heart and aorta full, when every obstacle to their emptying themselves appeared removed. This shows that there is a necessity for some degree of resistance from the arteries, that the heart may perform its office. As in other instances of arterial bleeding, the intestines were found free from flatus.

*9th. Experiment.* The abdomen of a dog was opened on the right side by a free incision: all the moveable intestines were protruded, but escaped unhurt. An incision, an inch in length, was made at the entrance of the renal veins. The flow of blood at first large, soon became less, and at length ceased: it was frequently renewed, but with less violence. The discharge was interrupted at a time when the heart continued to move regularly, and when the orifice of the vein was still open.

Towards the termination of life, the breathing became laborious, and was accomplished with the head raised and the mouth widely opened, and a weak involuntary howl was uttered. The pupils continued to contract for a long time, but at length they became insensible; the muscles of the eyes were spasmodically affected, as were the muscles of the breast and neck, especially the sterno-mastoideus. The face, limbs, and ears speedily became cold; and long before death, the breath was frigid.

On opening the cranium, great vascular turgescence was perceived in the membranes of the brain, and a deep red spot at the apex. The sinuses were all full. The surface of the brain was moist, and the ventricles contained a good deal of water.

The origins of the second, third, fourth, eighth, and accessory pair of nerves were in the state so frequently spoken of. Considerable redness of the upper part of the spinal cord was seen.

The nerves of the cervix, and upper part of the dorsum, resembled the greater part of the cerebral nerves; the rest were quite pale. The sinus venosi were full.

The left side of the heart was wonderfully hard, and contained clots of blood; the pericardium was very moist. Abundance of arteries and veins, full of blood, were seen on the mesentery and intestines. The mesenteric glands, and those of the neck, were very tumid. The right kidney was a third part larger than the left.

The death of this animal was the most tardy of any. The blood, on the vein being first opened, burst forth with great violence: Why, we may ask, was the death so slow, when, from opening the aorta and the jugular veins, the fatal event so rapidly took place?

The debility exceeded the ratio of the blood lost; for the bodies of those dogs which perished from arterial blood-letting, not only lost more blood, but were almost entirely deprived of it. A singular circumstance occurred during this experiment, viz. that the flow of blood ceased while the heart's motion continued uninterrupted. Here we may remark, how surprisingly the symptoms occurring during life accorded in every instance with the dissection. Were the eyes torpid, were their muscles convulsed, the optics, the third, fourth, and sixth pairs, severally and conjointly, were affected in the manner so frequently alluded to; were there at any time spasms of the neck and sides, the upper spinal nerves were found after death to have a net of red vessels spread round their origins, and serum was effused round them. The same may be said of all the other organs of the body and their nerves.

The summary of the conclusions to be drawn from these experiments will, it is hoped, in some measure answer the question proposed for solution; which is, Is there any difference between the effects of venous and arterial bleeding? and what is the precise nature of the difference?

The drawing blood from an artery diminishes more especially the quantity of venous blood; therefore arteriotomy is to be preferred when the veins are tumid.

The loss of arterial blood does not speedily disturb the respiration nor the heart's motion, nor does it rapidly break the strength; therefore, where we particularly wish to preserve entire the more important functions, let arteriotomy be had recourse to.

From arterial bleeding convulsions appear not apt to occur; therefore against such affections arteriotomy would most avail.

Blood let from veins does not particularly diminish the quantity of venous blood, but greatly disturbs respiration and the heart's motion, debilitates to a surprising degree, makes the veins every where turgid, and induces convulsions; therefore, when the pulsation is universally strong, as in every active inflammation, venæsection will be most serviceable; however, great caution is necessary in both, lest spasms be brought on.

If an excessive quantity of blood be lost, either from an artery or vein, water is effused within the brain; therefore, in drawing both, there is need of great caution, lest the tongue become cold, or the patient become sick, or his pupils dilated.

What are the symptoms of effusion of water within the brain?

In the removal either of arterial or venous blood, whenever the pulsations of the heart become very quick and feeble, the blood-letting should be stopped.

Thus, I think, we have learnt, that arterial and venous bleeding produce very different effects.

It is much to be wished that morbid dissections were carried on with the attention and zeal their importance so justly merits; we should then obtain a true knowledge of diseases, and consequently useful and comparatively certain methods of cure. Till the topography of diseases is established, till we ascertain their locality and individuality, and are able, as it were, to lay our finger on the focus of disorder, medicine will be always uncertain, always conjectural. If, as Dr. Saunders so justly and earnestly inculcates in his Lectures, all the phænomena of life (whether in health or disease) depend on the relation between the vascular and nervous systems, and that every living action, whether healthy or morbid, must be preceded by some change or changes in this relation, we have at once Ariadne's clue in our hands, which, if we follow its directions, will, IN TIME, liberate the medical profession from the endless maze of conjectures, doubts, and difficulties with which medicine is so much involved.

Why, it may be asked, has medicine made so little progress, compared with other arts and sciences? Is it not for want of fixed and definite principles of reasoning? Is it not evidently because there are few well understood and easily appreciable data upon which to build a solid superstructure? Can we wonder that systems, founded on gratuitous assumptions and partial limited views of Nature's operations, so rapidly rise and so rapidly moulder into dust? It cannot be too frequently repeated, that no medical opinion is of the smallest worth that clashes with correctly observed phænomena, either of health or disease.

How is a correct knowledge of the anatomical nature of diseases (if I may so speak) to be acquired? Is it not by patient and careful investigation of the symptoms of disease, compared and contrasted with an equally sedulous examination of *each and every principal organ of the body*, especially the brain, medulla spinalis, and their respective nervous cords, with great attention to the changes of relation between the vascular and nervous system throughout?

When the laws by which the motions of the heavenly bodies are well known and systematically arranged, why should we despair ofmedicine one day assuming that rank in the scale of sciences which its dignity and importance so imperiously demand?

In our times this is neglected, and we have abundance of speculation; but, alas! little accurate knowledge of the real nature of disease.

If any internal part suffers pain, how can he know what the nature of the disorder is, who is ignorant of the morbid changes which the viscera undergo? or how can a cure be effected by him who knows not what part is diseased? Nor can we admit it to be cruel, as many assert it to be, to promote the welfare of mankind in general by the destruction of a few animals.

THOMAS SEEDS, M. D.

*St. George's Square, Portsea.*

---

*To the Editors of the Medico-Chirurgical Journal & Review.*

GENTLEMEN,

Although there has been a short pause, or calm, in the contending opinions, relative to the fever named *Yellow* or *Bulam*; something like the delusive intervals of hope, so often seen in the latter stages of that formidable disease; which may induce the *contagional phalanx* into a belief, that all opposition to their doctrine must be at an end, and that any further attempts to controvert such powerful arguments, as have been brought forward, must prove abortive; I have, certainly with some degree of diffidence, endeavoured to muster my *quota* of countervailing argumentative forces, which will be shortly exhibited to public view, through the medium of the press.

These I should have some time past solicited the honor of marshaling under your banners, had I not considered they would be too numerous for so limited, yet so excellent a sphere of action, as your Journal undoubtedly is, for the exercise of individual opinion.

Through your valuable Miscellany, Gentlemen, I beg to announce, that I have in the press, which will shortly be published, a Series of Memoirs, on the cause and treatment of Yellow Fever, being the result of eight years experience in Jamaica, as assistant and regimental Surgeon, and that of the sickness at Cadiz, in 1810, as a Staff Surgeon: illustrated by cases and dissections, with a view to demonstrate that this disease was not in any degree contagious, in some of the instances stated to the public as being so, which fell within my own personal observations; and to corroborate, by my own and other well authenticated evidence, the important benefit resulting from early and copious venæsection in the treatment thereof.

Sir James Fellowes, in his Reports of the pestilential disorders of Andalusia, has not done me the honour to mention my name on the occasion, for very obvious reasons, which will be cursorily adverted to in the papers I am printing. I am, &c.

EDWARD DOUGHTY, Staff Surgeon.

19, *Goodge Street, April* 24th, 1816.

---

*Incomplete paralysis of the mouth and throat, from a wound penetrating the cranium; reported by Larrey.*—In the campaign of Moscow, a grenadier was struck by a Cossack's lance on the posterior part of the head, near the union of the occipital angle of the left parietal with the occipital bone. The iron traversed the substance of the bone without crushing it, and penetrated deeply into the posterior left lobe of the brain. He lay for some hours on the field, apparently dead; but was, at length, conveyed into the city. The sensitive faculties were long suspended.

The wound healed favorably; but the cicatrix was depressed; and there was a loss of substance of the bone, about four centimeters* in breadth, and one in depth: but the part was without pain, and the intellectual faculties unimpaired. It was, however, evident that injury had been inflicted on the nerves arising from the medulla oblongata and spinalis—as the glosso-pharyngeal, eighth, ninth, sub-occipital, and spinal.

The voice, at first hoarse and indistinct, was by degrees completely lost; deglutition difficult; taste and smell impaired. The muscles of the larynx were struck with partial paralysis; so that this organ is removed about two centimeters from its natural position: By this depression, the borders of the glottis are contracted; and this orifice covered by the epiglottis in consequence of the dragging of the *arytæno-epiglottidei* muscles. Hence, in order to respire in the erect posture, this man is obliged to keep the jaws constantly closed: thus, the larynx is drawn up by the simultaneous contraction of its elevator muscles and those of the jaw, as in frogs. Doubtless, like them, he would perish, if the jaws were kept long asunder.

The diaphragm shares this paralysis, and may be considered as not all at contributing to the functions of the lungs and larynx. It implicates also the pharynx, œsophagus, and stomach; for, in addition to the dysphagia, the stomach is completely insensible to the action of emetics.

No alternate movements of depression and expansion, isochronous with respiration, are observed in the abdomen. The man, when submitted to the most trifling experiments, feels great oppression, and exhibits signs of asphyxia: his visage becomes discoloured; his body covered with sweat; the habitual coldness of his extremities increased; the motions of the heart very slow, and, like the pulse, almost insensible. He respires better, and is much more at ease, in the horizontal position. Digestion is diffi-

---

* A centimeter is somewhat more than three lines. EDIT.

cult. He is compelled to eat little and frequently; and to live upon light aliment. He is greatly emaciated. Such are the phænomena attendant on this remarkable wound. *Journal de Médecine, par Leroux. Octobre*, 1815.

*Case of Blue Disease.*—The following appearances were observed by M. Ribes, on examining the body of a child, aged upwards of six years. It is singular that the signs of mal-formed heart were first developed during a convulsive attack induced by a contusion of the finger about his third year. The paroxysms of disordered circulation frequently recurred, and he suddenly expired in one of them. Externally, Skin of a brown ash colour; the lips, tongue, eye-lids, and finger nails bluish; toe nails less deeply tinged. *Abdomen;* liver and spleen of a deep hue; intestines brown; all the vessels injected with black blood; stomach somewhat dilated; the descending colon very much contracted. *Thorax;* thymus gland of considerable size; lungs dense, blackish, less voluminous than common; the right universally, the left partially, adherent to the costal pleura. The heart was of the natural size; rather shortened from base to apex, having the form of a cylindroid cone slightly flattened anteriorly, situated almost transversely, its base to the right and somewhat highest, its apex to the left and lower. The pericardium was thin, its internal surface lightly lubrified; the coronary vessels full of black blood. There were two ventricles and auricles, both the latter placed posteriorly; the right much dilated, and the foramen ovale unclosed, so as to admit a female catheter. This cavity received the venæ cavæ as usual, and communicated with the right ventricle. The left auricle, very small, received the pulmonary veins, and opened into its ventricle above and behind. Of the two ventricles, one was anterior, the other posterior. The former or right ventricle was large, its parietes very thick, and columnæ carneæ strongly marked. It was divided into two cavities by a double pillar of the tricuspid valve, fixed on one hand above and before to the floating margin of the valve, and on the other to the middle of the anterior wall of the ventricle; below and behind, to the middle of the posterior wall. This pillar was terminated at one of its borders by tendinous elongations, and allowed, by free spaces from above downward, a ready communication between both portions of the cavity. The ventricle, towards its base, was seen communicating above and behind with the right auricle; and anteriorly and to the left, continuous with the arch of the aorta, which was guarded at its origin by three sigmoid valves. Towards the left of the cavity, about ten lines from the origin of the aorta, was observed a small orifice, not more than three lines in diameter, leading to a canal about an inch long, which progressively diminished in size, and terminated in the pulmonary artery. This vessel, four times larger than the canal, was guarded at its origin by two sigmoid valves, one of more considerable size than the other, below the opening just mentioned. Between this and the origin of the aorta, another orifice was found, in diameter about six lines, unequally circular, and begirt by a kind of tendinous zone, on which the convex borders of the aortic valves were fixed. This orifice communicated with the left ventricle, which was placed at the posterior part of the heart, and not more than a fourth the size of the right ventricle; its parietes were thin, its columnæ carneæ, although small, strongly marked and numerous. Its base opened into the right ventricle by the orifice which existed between the origin of the aorta and the pulmonary artery. Posteriorly, and to the left, it opened also into the left auricle: the orifice of communication was guarded by the tendinous zone and two mitral valves; of which one was much the largest. "Thus," concludes M. Ribes, "the blood of the venæ cavæ, conveyed to the right auricle, passed on to the corresponding ventricle; a small quantity being transmitted to the left auricle by the foramen ovale. The great mass was propelled by the action of the ventricle into the aorta and thence into all parts of the body: but a small portion of the blood found its way into the pulmonary artery by means of a small opening in the left of the ventricle. This portion was conveyed to the lungs; returned by the pulmonary viens to the left auricle; from thence proceeded to the corresponding ventricle, and passed into the right ventricle to the aorta, by means of the orifice of communication which was placed at the origin of this artery."

*Bulletin de la Faculté de Médecine de Paris.*

Mr. JOHNSON lately opened a man who died of a disease very obscurely marked in some respects. He could lie on either side, and with the head low; his water was not particularly scanty, nor altered; he had short dry cough, without any expectoration; no fever, but appetite impaired, and bowels irregular. Towards the close of the disease, œdema of the ankles and hands, with some obscure symptoms of ascites. The thorax sounded badly, especially on the left side, where he complained occasionally of a dull heavy pain. He had been about nine months ill, and his first complaint was apparently pneumonia, from which he never properly recovered. He died rather suddenly and unexpectedly, though Mr. Johnson suspected hydrothorax. On opening the chest, the right lung was sound: the left was shrunk to the size of a large goose egg; in fact, there was nothing but the root of the lung, which was utterly incapable of performing any office. In the left cavity of the chest *nine pints* of water were found. The heart was not ostensibly affected, except that its structure was unusually flabby, pale, and easily lacerable. In the pericardium a few ounces of water.—This case shews how uncertain are the diagnostics in the strongest cases of disease. Nine pints of water in the chest, and yet the man could lie on either side with the head low; the urinary secretion little affected, and few or no startings from sleep.

*Method of decomposing solution of arsenious acid by the galvanic pile.*—"A tube of glass should be procured, open at both ends, After closing one of the extremities with a piece of bladder, a quantity of liquid arsenious acid is to be introduced. The tube is then to be placed in a vessel containing water slightly acidulated; into which must be brought the positive wire of the pile. The negative wire, terminated by a coloured metal, as gold or copper, must dip into the arsenious solution. At the end of twelve. fifteen, or twenty-four hours, the extremity of the negative wire will be seen covered by a white metallic crust, which is nothing but metallic arsenic. Sometimes, several days elapse ere this effect takes place. A pile of fifty pairs (of plates), one inch in diameter, reduced a solution containing but $\frac{1}{60}$ or $\frac{1}{40}$ of solid arsenious acid. The quantity of the acid is often so minute, that it is impossible to recognize the deposition. In such case, upon heating the negative wire after the operation, the smell of garlick, which characterizes arsenic will be perceived. Jæger, who first proposed this method of decomposing arsenious acid, sometimes failed in the process; probably, according to Fischer, because he brought both wires into the tube containing the arsenical solution." *Toxicologe generale, par Orfila. Tome Premier.*

---

The following Report of the London Vaccine Institution, read at the late Annual Meeting of the Governors, has recently been printed and circulated.

"Whenever a Society is engaged in an arduous, highly important undertaking, having to combat the prejudices attached to former usages, as well as those of ignorance and selfishness, (and such, it must be admitted, is the case of the London Vaccine Institution, aiming at the suppression, and, if possible, the extinction of the Small Pox,) it cannot be matter of small exultation, if they should be able, at a certain stage of the contest, to announce any remarkable advantage gained upon the enemy, inspiring hopes of complete victory. That this is in some measure the case, by the efforts of this Institution since the last annual Report, it is believed will be admitted, when the Governors are informed, that about one hundred additional vaccinators of the medical profession, many very eligibly situated in the most poor and populous parts of the metropolis, have been added to the effective force of the establishment during the last year; making the total number of inoculators in the metropolis and its environs to exceed two hundred. A considerable number of medical practitioners have also been appointed inoculators in many populous cities and towns in the United Kingdom and Abroad. And herein ought to be inscribed, as a lasting memorial to the honour of a liberal and most useful profession, the sense of respect and gratitude the Managers feel for such a sacrifice of gratuitous services in this great cause of humanity and benevolence.

"It appears, that during the last year,

| | |
|---|---|
| There have been Vaccinated by Dr. Walker . . | 2,351; |
| From the beginning . . . . . . . . . . . | 23,711. |
| By the appointed Inoculators in the Metropolis . | 9,893; |
| From the beginning . . . . . . . . . . . | 28,887. |

By the appointed Inoculators in the Country . . 16,457;
From the beginning . . . . . . . . . . . 324,007.

Dr. Walker, since the last Report, has supplied to 3,253 applicants, 16,265 charges of matter; from the beginning, to 36,631 applicants, 175,768 charges.

"After such an exposition of facts, the friends of Vaccination need not query whether the cause is gaining ground.

"It may not now be improper to take a short retrospective view, of some of the great advantages the public have derived from the introduction of the prophylactic practice of Vaccination.

"In ten years preceding the æra of the establishment of Vaccination in the metropolis, viz. 1793 to 1802, inclusive, the deaths by Small Pox, as reported by the annual bills of mortality, amounted to 18,202 persons, being 1820 average deaths per annum. In the last two years the deaths reported are 1363, being full 681 per annum. Whence it appears the mortality has been lessened two-thirds by means of Vaccination, although the metropolis has indubitably kept increasing since 1802. This is no small triumph in favour of the protecting practice; and it is presumed the London Vaccine Institution can fairly lay in its claim for affording a considerable share of this great benefit to the public, seeing it has always had a much greater number of practitioners engaged in the cause, than all the other Vaccine Institutions conjointly. Nevertheless, the Board of Managers cannot forbear expressing their deep regret, that the mortality, though comparatively reduced, is still so great as it is; and they wish to draw a faithful picture of this remaining evil, believing it is but imperfectly understood to what an extent it still reaches. The friends of Vaccination can never view, but with alarm, the existence of so insidious an enemy as the Small Pox, in any populous town; but, above all, its ravages are to be particularly deplored in the metropolis, having such continual intercourse and communication, not only with the whole of the United Kingdom, but with the world at large. Though the number of deaths stated by the annual bill is 725, it means buried in the church-yards only; it does not include those in other burial grounds, which are not returned to the parish clerks' office, and which are probably to a larger amount. From the populous districts of Marybone, Pancras, and Kensington, not being included in the present bills of mortality, no returns of their deaths are ever made. It will therefore appear, on a very moderate calculation, that not less than 2000 persons fell victims to the Small Pox during the last year. But the existing evil does not stop here. It is well known, that where 2000 persons have died, five times that number must also have endured this dreadful disease. Hence, not only 2000 persons have died by the Small Pox, but 10,000 other persons must have suffered the pains of this malignant pestilence, though escaping with life. To describe the miseries, sufferings, and disadvantages, accruing from such a scene, would require more detail than the summary nature of a Report will allow: but the Managers hope this statement of the real extent of the evil will not fail to arouse fresh efforts to repress or extinguish it; and they are encouraged in this expectation by the known practicability of checking this pestilence, as evinced in the populous cities of Vienna and Milan, where it has been completely subdued by municipal regulations and magisterial vigilance.

"At a time when the Inoculation of the Small Pox is ceasing altogether at the Small Pox Hospital; when His Majesty's Attorney General is happily succeeding in the Court of King's Bench, in restraining, or severely punishing, the private practitioners, propagating the deleterious disease in the metropolis, the Managers would act unworthily of the spirit of the Institution, if they did not use every effort to carry conviction to those who hesitate, as well as to afford them the means of protection. During the last year, they have sacrificed upwards of 500*l.* beyond their ordinary expenses, in endeavouring to inform the public mind, throughout the country as well as in the capital. The pamphlets and other papers of the Society, with plenteous supplies of vaccine matter, have been gratuitously forwarded to upwards of a thousand different stations throughout the island. They have been distributed most extensively throughout the capital, to the amount of many thousands, as well as to various places abroad; even to parts not within the confines of the Bristish empire. A Black from Boston in Massachusets, Prince Sanders, a gentleman of high character as well as talent, has received not only instructions in vaccination, but has been well exercised in the practice of it, at the principal stations of the Institution, and been furnished with large supplies of the guardian fluid. He has set out to St. Domingo, in hopes of bearing the benefits of Vaccination, as well as all the consolations which the kind messages, through him, from the most eminent members of the African Institution, will waft to the sable inhabitants of that fine island. The institution has already appointed Inoculators in the British trans-atlantic colonies.

"In these extra exertions of the Society, the Managers will not reckon in vain on the continued support of the Governors. The name and influence of these will continue to give success to their most earnest applications for aid, wherever they may venture to address their solicitations in behalf of the great charity. Indeed, in every rank of society they have to acknowledge a liberality which animates them to extraordinary exertions; and they trust that the continued encouragement of a generous public, will eventually enable them most effectually to contribute their share to the universal extinction of the Small Pox—the extensive pestilence of the last twelve centuries; the desolating disease unknown to the Hebrews, the Greeks, and the other nations of antiquity and which it is to be hoped will shortly become utterly unknown, except as recorded in the page of history.

| | | | |
|---|---|---|---|
| "The Rceipts of the Institution, since formation, in 1806, amount to . . . . . . . . . . . . . . | £.4765 | 7 | 2 |
| Its Disbursements, to . . . . . . . . . . | £.4662 | 5 | 0 |
| Balance remaining on hand . . . . . . . . | £. 103 | 2 | 2 |

A Meeting of Army and Naval Surgeons was lately held in the Metropolis, when it was resolved to petition Parliament for a repeal of the obnoxious clauses in the late Apothecaries Act, by which these Gentlemen are prevented settling themselves as Practitioners in any Part of England or Wales, without examination by and licence from the Apothecaries Company. The following is the spirit of the Resolutions unanimously agreed to at that Meeting, viz.

"That the Meeting do feel justly aggrieved by the conduct of the Company of Apothecaries of London, in consequence of the operation of the Apothecaries' Act, passed last Sessions of Parliament, by which the whole of the Medical Officers of both services are prevented from commencing practice, although they have been found qualified by Boards of Examiners appointed by the Legislature, to compound medicines, to visit and prescribe for the sick.

"That the Meeting do consider a late Advertisement from the Master and Wardens of the said Company of Apothecaries, inserted in several public Papers, giving information, "that on any person producing his commission or warrant as Army or Navy Surgeon, prior to the 1st of August last, he should be considered as having furnished proofs of a sufficient medical education to admit him to an examination for a certificate to practise as an Apothecary," to be a direct insult to the whole of the Army and Navy Medical Officers, and calculated to depreciate their professional qualifications in the eyes of the public, and that they are firmly resolved to endeavour at procuring through the Legislature, a repeal of such clauses of the Apothecaries' Act as can be construed to their exclusion from practice.

Dr. Clutterbuck will begin his Summer Course of Lectures on the Theory and Practice of Physic, Materia Medica, and Chemistry, early in June.

Died.—At his house, Laurel Lodge, Twickenham, aged 42, Thomas Terry, M. D.—In George Street, Edinburgh, Dr. Thomas Hay.—At Brompton, Kent, Mr. Richard Morris, Surgeon. —On the 15th of April last, at Haslar Hospital, Dr. William Bickley, late one of the Surgeons of that establishment. He was a man of very active mind, and considerable talents in his profession, and was selected by Lord St. Vincent to serve under his flag. His death was caused by diabetus mellitus, which the Doctor himself did not suspect till within a month of his dissolution. About a fortnight before that event, Watts's plan was adopted; but the quantity of water was not diminished, while the debility was increased. After two pretty copious venæsections, the plan was given up, and in a few days he sunk under the affliction.

